# Flexible work arrangements in open workspaces and relations to occupational stress, need for recovery and psychological detachment from work

**DOI:** 10.1186/s12995-020-00258-z

**Published:** 2020-03-19

**Authors:** Stefanie Mache, Ricarda Servaty, Volker Harth

**Affiliations:** 1 Institute for Occpuational Medicine and Maritime Medicine (ZfAM), University Medical Center Hamburg-Eppendorf (UKE), Hamburg, Germany; 2 Rosenheim University of Applied Sciences, Rosenheim, Germany

**Keywords:** Flexible work, Mental health, Open workspaces, Working conditions

## Abstract

**Background:**

The trend is going into the direction of flexible work arrangements in open workspaces in which employees can decide where and when to work. The aim of this study was to analyze effects of a transition to open workspaces including Activity Based Working (ABW) on employees’ working conditions and their levels of occupational stress, need for recovery and psychological detachment from work.

**Methods:**

Employees of a large technology company responded to a baseline and two follow-up measurements over one year. Data were collected via online survey assessing the employees’ mental demands, workload, job autonomy, support from supervisor, team collaboration, satisfaction with communication climate and three well-being outcomes (occupational stress, need for recovery and psychological detachment from work). Descriptive statistical analyses, analyses of variance and regression analyses were applied to test the hypotheses.

**Results:**

Significant differences in working conditions were found after the transition, e.g. reduced mental demands, but an increased workload. Job autonomy, team collaboration and satisfaction with communication climate increased. Levels of occupational stress decreased significantly over time. Regression analyses revealed substantial associations between flexible work arrangements, job resources and occupational stress.

**Conclusion:**

The results contribute to the current knowledge on flexible work arrangements in open work spaces. They can be used to design future work settings aimed at increasing employees’ well-being and job performance. Further practical implications and recommendations for future research are discussed.

## Background

With the rising popularity of new forms of working, the scientific interest in flexible work arrangements such as “Activity Based Working” (ABW) and the design of office spaces is increasing as well. ABW is defined as a work design in which employees can control and organize the timing and place of their work to a great degree [1]. They can choose where they work, including the office, home, and during commuting time (e.g., in train). The key design feature of ABW is the usage of open work offices with a variety of shared work spaces designed for different work tasks, so-called “activity-related workspaces” (e.g., focus and concentration rooms, meeting rooms etc.) which employees can use depending on the nature of their work activities (e.g. meetings etc.). Work spaces are chosen and used by employees based on their current work tasks with regular transitions between them [2]. These flexible work arrangements also include the principle of “desk-sharing”, which means the employees do not have an assigned workstation anymore and can choose their workspace freely. The work environment is often divided into so-called neighborhoods, which ensure that colleagues from the same department still have proximity to one another. In addition, employees in ABW settings use various information technology (e.g. personal laptops and smartphones) [2, 3].

Recently published research studies did not show whether ABW has more advantages or disadvantages [2]. A review by Engelen et al. summarized that ABW has positive effects in the areas of interaction, communication, control of time and space, and satisfaction with the workspace; negative effects on concentration and privacy [2–4]. To date, there is a gap of studies examining relations between the concept of ABW and employees’ well-being outcomes such as occupational stress and need for recovery [5, 6].

Prior studies have shown that allowing employees to manage their own working hours, choose their work location, and organize their tasks with reduced job control increases employees’ perceived job autonomy [7, 8]. This working conditions may allow employees to vary job-specific requirements and needs [3, 9]. Therefore, it is suggested that ABW offers employees a very high level of autonomy. In addition, the present study assumes that this increased autonomy in choosing optimal working conditions reduces the degree to which employees perceive their job as mentally demanding.

Contradicting results exist for social relations in ABW. Some studies found that ABW offers more opportunities for communication [1, 10]. In contrast, other studies reported social and professional isolation, reduced feedback, and lack of support from supervisors and colleagues [8, 11, 12]. It is reported that the open office type offers fewer opportunities for face-to-face interactions and for giving and receiving support [13]. In consequence, sharing information and knowledge could be more demanding and may have a negative influence on perceived workload. In sum, we predict differences in supervisor and co-worker support after a transition to ABW.

The Job Demands-Resources (JD-R) model by Demerouti, Bakker, Nachreiner, & Schaufeli (2001) builds the theoretical framework for this study. The JD-R was chosen as an alternative to other models of employee well-being, such as the Job Demand-Control model (JDC model) and the Effort-Reward Imbalance model (ERI). The JD-R model includes a wide range of working conditions into the analyses of organizations and employees in comparison to a given and limited set of predictor variables that may not be relevant for this kind of work setting (such as in the ERI model). Furthermore, instead of focusing solely on negative outcome variables (e.g., burnout) the JD-R model includes both negative and positive indicators and outcomes of employee well-being [14].

Due to its flexibility, the model can be applied to a diverse range of different professional and operational contexts (such as ABW). The foundation of the JD-R model is that all factors, which are correlated with job stress, can be categorized in job demands and resources [14]. A key proposition of the JD-R model is that interactions between job demands and resources are important since certain job resources can buffer negative effects of psychological stress [15]. It is known that changes in work organization may implicate significant changes in job demands and resources. In relation to the JD-R model [15], changing job characteristics in case of ABW may also impact the employees’ perceived occupational stress and well-being [14]. We assume that alterations in work arrangements such as ABW and increased job autonomy should have a beneficial effect on levels of employees’ perceived stress.

ABW may also influences the way in which individuals perceive their need for recovery. The concept of need for recovery was deduced from the Effort-Recovery theory model [16]. In this model work produces costs in terms of effort during the working day. Effort results in an array of emotional, cognitive, and behavioral symptoms that are reversed when the effort stops. Need for recovery is characterized by temporary feelings of overload, irritability, social withdrawal, lack of energy for new effort, and reduced performance [16]. We assume that ABW may create optimal and flexible working conditions. So, each employee has the autonomy and freedom of choice to decide how and when to work to achieve the best outcome. The increased flexibility and the freedom associated with organizing one’s own time and job practices under diminished supervisory control may also positively affect employees’ need for recovery.

Moreover, we focus on psychological detachment from work that is defined as the ability to mentally disengage oneself from work when being away from the workplace, and is considered as a strong indicator of psychological recovery [17–19]. In the Extended Stressor-Detachment model psychological detachment is proposed as a powerful mechanism in the link between stressors and strain reactions. The model assumes that job stressors hinder psychological detachment from work. Previous research studies have provided substantial empirical support for the model by showing negative associations between job stressors (e.g., mental demands, workload, time pressure) and psychological detachment from work [17, 18, 20]. We assume that within ABW self-perceived job autonomy and flexibility in working time and location which are predicted to increase - can buffer negative effects of job demands (e.g., high levels of workload) on psychological detachment from work.

The aim of this research study was to investigate whether a transition from a normal office work setting to flexible work arrangements in open work spaces involves differences in working conditions (e.g., job demands and job resources). In addition, a further focus is on relations between flexible work arrangements in open work spaces and employees’ perceived occupational stress, need for recovery and psychological detachment from work.

For this study, we predict that:

- *Hypothesis 1: Levels of perceived job demands (*i.e. *mental demands and workload) are significantly lower after the transition to open work spaces including ABW.*
- *Hypothesis 2: Levels of perceived job resources (*i.e. *job autonomy, support from supervisor, team collaboration and communication) are significantly higher after the transition to open work spaces including ABW.*
- *Hypothesis 3a: Levels of occupational stress are significantly lower after the transition to open work spaces including ABW.*
- *Hypothesis 3b: Levels of need for recovery are significantly lower after the transition to open work spaces including ABW.*
- *Hypothesis 3c: Levels of psychological detachment from work are significantly higher after the transition to open work spaces including ABW.*
- *Hypothesis 4: Flexible working arrangements in open work spaces are significantly negatively related to occupational stress and need for recovery and positively related to psychological detachment from work.*
- *Hypothesis 5: Job autonomy moderates the relationship between flexible working arrangements in open work spaces and occupational stress, need for recovery and psychological detachment from work*.

## Materials and methods

### Study design and setting

The study was designed as a longitudinal study, because differences over time should be shown. Data collection took place in 2017/2018 from employees of a large technology company. A transition to open work spaces including ABW was planned for the head quarter. Consistent with the principles of ABW, the main organizational changes were implemented. The transition has been communicated to the employees.

Three data measurements were administered via online surveys. The first survey (T0) was distributed one month before changing the workplace into open work spaces including ABW. The second survey (T1) was circulated three months after the change and the third wave of data (T2) one year after the change.

### Study sample and recruitment of participants

All employees working in ABW with flexible open workspaces were recruited. Work tasks included administrative and finance related tasks. Recruitment was performed via email and/or direct communication to contribute to this study.

Employees were included in the study based on the following criteria: (1) office representatives, (2) working full time, (3) working experience with at least one year of service, (4) German speaking employee. Every participant gave written informed consent. Employees were excluded from the study based on the following criteria: 1) employees working part-time, 2) trainees, 3) employees, who do not work in the activity based work environment.

The German Headquarter in Hamburg comprises 203 employees. Out of the 121 eligible participants contacted (all employees working in the office implementing ABW), 103 employees completed the first questionnaire (T0; 81.4% response rate). The response rate of the first follow-up wave (T1) was 78.2%, and the second follow-up wave (T2) had a 72.8% response rate. Participants were only included if they had provided data for all three assessments (*N* = 71 employees, 59% of the initial group). No significant differences could be found between responders and non-responders in the first or second follow-up survey with regard to socio-demographic or work-related variables.

#### Survey procedure

Employees were requested to complete an online questionnaire before moving in open work spaces (baseline), after 3 months (follow-up survey 1) and after 12 months (follow-up survey 2). The surveys were conducted by using a secure web-based survey system, via links within e-mail messages.

After receiving the signed consent form from the physicians, the link for the survey was sent to each participant. A link for the follow-up survey 1 was emailed three month after the transition, the link for the follow-up survey 2 was emailed twelve months later.

### Measurement

All participating employees were asked to complete three online surveys (before transformation and two follow-up surveys). All measurements were conducted as self-reports, using a secure and widely used external online survey collection service. Links to the surveys were e-mailed directly to the employees. All surveys contained anonymous data only.

#### Flexible work arrangements

According to van Steenbergen et al. (2018), Activity Based Working was assessed by using five items: (a) “I decide for myself where (office, home, elsewhere) and when I work,” (b) “I do not have my own personal desk (flex-desk concept)” c) “In the office, I work in an ‘activity-related’ manner (e.g., using spaces for concentration, communication, meetings)” d) “I use information technology (e.g., smartphone, laptop), so I can work at any chosen location or time,” (e) “I regularly work remotely with my colleagues”. Items were scored on a 5-point Likert scale ranging from 1 (=totally disagree) to 5(=totally agree) [19]. The Cronbach’s alpha coefficients of the ABW scales were 0.78 (ABWT0), 0.80 (ABWT1), and 0.79 (ABWT2), respectively.

#### Job demands

Mental demands were assessed using the Copenhagen Psychosocial Questionnaire (COPSOQ I) [21]. Items were scored on a 5-point Likert scale ranging from 1 (= always) to 5 (= never). An example of the item is: “My work requires a high level of concentration” [21]. Reliability and validity of the COPSOQ I are good [21].

Workload was measured with 4 items from the Kurzfragebogen zur Arbeitsanalyse (KFZA) instrument [22]. A sample item is: “I have too much work”. Items were scored on a 5-point Likert scale ranging from 1 (=very little) to 5 (=very much). Cronbach’s alpha coefficients ranged between 0.75 and 0.86.

#### Job resources

Job autonomy and team collaboration were measured using a 3-item scale of the KFZA instrument, e.g., “Can you decide for yourself how you execute your work?”. Items were scored on a 5-point Likert scale ranging from 1 (never) to 5 (=always). The reliability coefficients were 0.81 (T0), 0.79 (T1), and 0.81 (T2). Coworker support was assessed with three items of the KFZA including “Can you ask your colleagues for feedback if necessary?” (1 = never, 5 = always; alpha coefficients were 0.78 (T0), 0.80 (T1), and 0.75 (T2)) [22].

Supervisor support was assessed with 3 items of the COPSOQ, such as “Can you ask your supervisor for help if necessary?” (1 = never, 5 = always; alpha coefficients were < .80 at all three measurements).

A 5-items scale of the Communication Satisfaction Questionnaire (CSQ) was used to measure changes in satisfaction with communication climate and supervisory communication [23, 24]. Items were used with a Likert type scale ranging from very satisfied (1) to very dissatisfied (7). As an example: “My supervisor offers guidance for solving job-related problems.” The questionnaire shows good psychometric properties [25]. Alpha coefficients ranged between 0.73–0.82.

#### Well-being outcomes (occupational stress, need for recovery, psychological detachment)

Occupational stress symptoms were measured using the 4-item subscale of the COPSOQ II [26]. Items were scored on a 5-point Likert scale ranging from 1 = not at all to 5 = all the time. An example item is “In the last month, how often have you been upset because of something that happened unexpectedly?” Research supports the psychometric qualities of the scale [26]. Alpha coefficients were 0.75 (T0), 0.73 (T1), and 0.76 (T2) [27].

Need for recovery (NFR) was measured by using the German version of the NFR after work scale that included 11 items on a dichotomous (yes/no) scale [27]. Typical items of this scale are: “Because of my job, at the end of the working day I feel rather exhausted”, “I find it hard to relax at the end of a working day”. The sum score is calculated by adding the individual’s scores on the 11 items. Higher scores indicate a higher degree of need for recovery after work [27].

Psychological detachment from work was assessed with the same named 4-item subscale from the Recovery Experience Questionnaire [28]. The scale ranges from 1 (= I do not agree at all) to 5 (= I fully agree), with higher scores indicating higher psychological detachment. An example item is: “During leisure time, I forget about work.” The scale shows good psychometric properties [28]. Alpha coefficients were 0.80 (T0), 0.78 (T1) and 0.79 (T2).

Sociodemographic variables included age, gender, years of working experience, job position, marital status and presence of children (see Table 1).

**Table 1:** Sociodemographic of the study sample

Variables	***N***	%
Gender (*n* = 71)		
Female	38	53.5
Male	33	46.5
Age (*n* = 71)		
≤ 29 years	13	18.3
30–39 years	33	46.5
40–49	17	23.9
≥ 50 years	8	11.3
Relationship status (*n* = 70)		
Single	15	21.4
In a relationship	55	78.6
Children (*n* = 70)		
Yes	37	52.9
No	33	47.1
Work experience (*n* = 71)		
1–2 years	16	22.5
3–5 years	27	38.1
more than 5 years	28	39.4
Job position level (*n* = 71)		
Assistant	13	18.3
Associate	23	32.4
Specialist	28	39.4
Manager	7	9.9

Note: Sample size differs between *n* = 70 and *n* = 71 due to missing data

### Statistical analyses

When variables were measured by multiple items, scale means were calculated and used in all analyses. All items relevant for hypotheses testing were continuous variables. For regression analyses, especially for moderation analyses, all variables were centered at their means to address potential problems of multicollinearity [29].

The data were tested for normal distribution. Normal distribution can be identified by looking at skewness and kurtosis values, histograms or statistical tests [30]. All of this output was generated and interpreted, but concrete confirmation of the normality assumption in numbers was tested on the basis of the Shapiro Wilk test. In contrast to the Kolmogorov-Smirnov test, the Shapiro Wilk test is adapted for small sample sizes [31]. The Shapiro-Wilk-Test indicated that data was normally distributed (*p* = .32), skewness and kurtosis of the variables were mainly beyond the threshold of < 1.0. Therefore, we used parametric tests.

A check for outliers was conducted by creating box-and-whiskers plots. The whiskers of the plots showed no outliers.

We analyzed the differences of the transition in working conditions (job demands and resources) as well as on the outcome variables by using repeated measures of univariate and multivariate analysis of variance (RM ANOVA, RM MANOVA) (hypotheses 1–3). In order to further examine the significance and magnitude of the effects of time, we inspected post-hoc multivariate effects of time and conducted pair-wise comparisons. In addition, we performed Bonferroni corrections due to multiple testing. Effect sizes (d) were interpreted as follows: 0.01–0.059 = small; 0.059–0.138 = medium; and ≥ 0.138 = large effect [32]. A Pearson correlation matrix was conducted to see the relations between flexible working arrangements, job demands, job resources and the three well-being outcomes (occupational stress, need for recovery and psychological detachment) (hypothesis 4). No excessive correlations (all r’s < .60) were analyzed (see Table 3). Therefore, we conclude that multicollinearity does not occur here. The results of the Mauchly’s tests of Sphericity show that sphericity has not been violated (*p* > .05).

Multiple regression analyses were used to examine the relationship of the predictors (flexible work arrangements, job demands and resources) and occupational stress, need for recovery and psychological detachment as an outcome (hypotheses 4, 5). For stepwise regression analysis a sample size of *N* = 71 would be too small [29]. For moderation analysis, all variables were centered at their means to prevent potential problems of multicollinearity [29] and make lower-order effects interpretable [30]. R^2^ was measured to examine the amount of variability in the outcome which could be explained by the predictors.

Regarding the moderating effect of job autonomy on the linkages between flexible working arrangements and the three well-being outcomes, a moderated multiple regression analysis was performed (hypothesis 5). The flexible work arrangement scale and job autonomy scale were centered at their means before computing the interactions. In addition, a simple slopes analysis was performed. We controlled for the influence of sociodemographic variables. We used standardized regressions weights (β) to assess the strengths of association between the variables. Based on Cohen’s recommendations [73], β = 0.1 was interpreted as a weak, β = 0.3 as a moderate, and β = 0.5 as a strong association. We defined *p* < 0.05 as level of significance. All provided *p*-values were two-tailed. Data were calculated using the SPSS® software package for social sciences; Version 23.0.

Statistical analyses were carried out using IBM SPSS Statistics for Windows V.23.0 (IBM Corp; released 2015; Armonk, New York, USA).

## Results

### Descriptive statistics

Employees’ sociodemographic characteristics at baseline are given in Table 1. Half of the respondents were female (53.5%); 78% were in a relationship and 52.9% had children, mean age was 39 years (SD = 9.5 years). The majority of the participating employees worked for more than 5 years in the current position (39.4%).

Means and standard deviations for differences in flexible work arrangements, job demands, job resources and well-being outcomes are presented in Table 2.

**Table 2:** Differences in means and standard deviations for job demands, job resources, and well-being outcomes before and after the transition to open work spaces

Variables	T0		T1		T2		Effect time
	M	SD	M	SD	M	SD	*p*
Flexible work arrangements (scale)	3.54^1,2^	1.35	5.14^0^	1.69	5.09^0^	1.62	.00
Desk sharing	3.58^1,2^	1.47	5.04^0^	2.01	5.13^0^	2.14	.00
Activity based working	3.84^1,2^	1.95	5.89^0^	1.87	5.93^0^	1.94	.00
Flexibility in Time and Location	3.38^1,2^	1.28	4.58^0^	1.94	4.71^0^	1.89	.00
Information Technology	4.01^1,2^	1.79	5.01^0^	2.01	5.63^0^	2.14	.00
Working outside the office	3.89^1,2^	1.84	4.87^0^	2.17	4.91^0^	2.21	.00
Job demands							
Mental demands	3.95^1,2^	1.15	3.78^0^	1.21	3.41^0^	1.24	.02
Workload	3.68^1,2^	1.04	4.02^2^	0.98	4.17^1^	1.09	.02
Job resources							
Job autonomy	3.48^1,2^	1.12	3.71^2^	1.06	3.96^1^	1.01	.03
Collaboration team	4.01^2^	1.27	4.10^2^	1.22	4.51^1^	1.25	.04
Support supervisor	3.78^ns^	1.02	3.71^ns^	1.03	3.75^ns^	1.02	.09
Supervisory communication	4.71^ns^	1.69	4.58^ns^	1.61	4.67^ns^	1.65	.08
Communication satisfaction: climate	5.68^ns^	1.51	5.95^ns^	1.47	6.28^ns^	1.53	.19
Well-being Outcomes							
Occupational stress	3.87^2^	1.05	3.61^ns^	1.08	3.51^0^	1.01	.01
Need for recovery	4.85^ns^	1.02	4.71^ns^	1.03	4.69^ns^	1.09	.61
Psychological detachment from work	3.11^ns^	1.10	3.19^ns^	1.06	3.20^ns^	1.09	.86

Note. 0 significantly differs from pre-measure, 1 significantly differs from post-measure 1; 2 significantly differs from post-measure 2; *ns* no significant differences from other measures; *p* values for univariate analyses

We conducted a RM MANOVA to check whether flexible work arrangements (e.g. ABW) were higher at T1 and T2 than at T0. The results of the combined flexible work arrangements showed a significant increase over time *F* (10,285) = 39.94, *p* < 0.01. The univariate analyses showed significant effects of time on the variables desk sharing, activity based working, flexibility in time and location, information technology use, working outside the office (see Table 2).

### Differences in job demands after the transition to open work spaces

Lower levels of perceived job demands (workload, mental demands) were assumed in Hypothesis 1. The RM ANOVA showed a significant effect of time on workload (*F* (2,210) = 4.16, *p* = .02; d = .03). Pairwise comparisons showed that workload was significantly higher a year after the transition (T2) (M = 4.17, SD = 1.09) than before the transition (T0) (M = 3.68, SD = 1.04), *p* < .05.

The RM ANOVA exposed a significant effect of time on mental demands (*F* (2,210) = 3.75, p = .02; d = .03). Pairwise comparisons showed a significant decrease in mental demands a year after the transition (T2) (M = 3.41, SD = 1.24) than at T0 (M = 3.95, SD = 1.15), *p* < .01, d = .05.

All in all, hypothesis 1 was partly supported, in that we found significant reduced levels of mental demands after the transition to open work spaces.

### Differences in job resources after the transition to open work spaces

We also tested Hypothesis 2 to examine effects of the transition on the job resources (autonomy, supervisor support, team collaboration, communication, etc.).

Job autonomy showed effects on time *F* (2,210) = 3.61, *p* = 0.03, d = .03). A pairwise comparison specified that job autonomy was significantly higher at T1 (M = 3.71, SD = 1.06; *p* < .05), and at T2 (M = 3.96, SD = 1.01; p < .05, than at baseline (T0) (M = 3.48, SD = 1.12).

In addition, we examined effects on time for differences in collaboration between colleagues after a change to ABW *F* (2,210) = 3.24, *p* = 0.04; d = .03) (hypothesis 2c). Team collaboration was rated significantly higher a year after the change (T2) (M = 4.51, SD = 1.25) than before the change (T0) (M = 4.01, SD = .1.27; *p* < .01).

We also tested whether levels of supervisor support, communication climate and supervisory communication differ after a transition to ABW. Univariate results revealed no significant differences (see Table 2).

In sum, hypothesis 2 can be partly confirmed.

### Differences in occupational distress, need for recovery and detachment from work after the transition to open work spaces

In addition, we found a significant decrease for occupational stress after the transition at T2 (*F* (1,140) = 6.47, *p* < .01; d = .04) (see Table 2). The results of one-way repeated variance analyses showed no significant differences in psychological detachment from work and need for recovery (*p* > .05). So, only hypothesis 3a was supported.

### Relations between flexible work arrangements (ABW) and employees’ well-being outcomes

A Pearson correlation matrix was performed in order to check the correlations between flexible working arrangements, the three dimensions of employee well-being and job autonomy (hypotheses 4). Table 3 shows the correlations between the variables. There are significant correlations (*p* < .05) between the different dimensions. In detail, we found a significant negative correlation between levels of flexible work arrangement and perceived occupational stress (*r* = −.19, *p* < .05) as well as for need for recovery (*r* = −.21, *p* < .01) and a significant positive correlation for psychological detachment (*r* = .15, *p* < .05). The correlation matrix shows that there is no significant correlation between gender and the variables used. In addition, there is a negative correlation between age and occupational stress (*r* = −.21, *p* < .01) and a positive association between age and need for recovery (*r* = .25, *p* < .01).

**Table 3:** Correlations between working conditions and well-being outcomes

Independent variables	Dependent variables		
	Occupational stress	Need for recovery	Psychological detachment from work
**Age**	−.21**	.25**	.10
**Work experience**	.15*	.11*	.15*
**Flexible work arrangements (ABW)** (scale; T2)	−.19*	−.21**	.15*
“I do not have my own personal desk (flex-desk)”	.21*	.17*	.07
“I decide for myself where (office, home, elsewhere) and when I work”	−.25**	−.18**	.14*
“I use information technology (e.g., smartphone, laptop), so I can work at any chosen location or time”	.17*	.11*	−.20**
“I regularly work remotely with my colleagues”	−.12*	−.18**	−.09
“In the office, I work in an ‘activity-related’ manner (e.g., using spaces for concentration, communication, meetings)”	.14*	.16*	.09
**Mental demands** (T2)	.30**	.36**	−.19**
**Workload** (T2)	.39**	.31**	−.25**
**Job autonomy** (T2)	−.16*	−.21**	.08
**Collaboration team** (T2)	−.21**	−.19*	.11
**Support supervisor** (T2)	−.17	−.10	.14*
**Supervisory communication** (T2)	−.19*	−13*	.10
**Communication satisfaction: climate** (T2)	−.11	−.09	.09

The control variables correlated only with a few of the independent and the dependent variables. Only age and work experience correlated with the three well-being outcomes. Therefore, they were used as control variables in the regression analysis. According to the regression analysis (see Table 4), there was indeed a significant negative relationship between flexible working arrangements and occupational stress (β = −.19, *p* < .01). In accordance with the regression analysis, flexible working arrangements were negatively related to the need for recovery (β = −.15, *p* < .05). Referring to the multiple regression analysis, there is also a positive relationship between flexible working arrangements and psychological detachment. Therefore, hypothesis 4 was confirmed.

**Table 4:** Results of multiple regression analysis for the interaction effects on occupational distress, psychological detachment from work and need for recovery

	Occupational stress			Need for recovery			Psychological detachment from work		
	Step 1	Step 2	Step 3	Step 1	Step 2	Step 3	Step 1	Step 2	Step 3
Variable									
Age	−.17*	−.15*	−.14*	.19*	.18*	.16*	.08	.06	.05
Work experience	−.11	−.09	−.08	−.09	−.06	−.05	.14*	.12*	.12*
Flexible work arrangements (ABW)	−.19*	−.18*	−.17*	−.15*	−.13*	−.11*	.10	.09	.08
Job autonomy		−.16*	−.14*		−.19*	−.17*		.08	.05
ABW x job autonomy			.12*			−.06			.07
R^2^	.28	.33	.39	.16	.18	.20	.10	.14	.15

Note: The coefficients are standardized ß weights. *p < .05; **p < .01

Hypothesis 5 predicted interaction effects of job autonomy on the relationship between flexible working arrangements and the different dimensions of employee well-being. The last step in Table 4 shows that the interaction term between flexible working arrangements and job autonomy was significant for occupational stress (ß = .12, *p* < .05). The results revealed that the relationship between flexible working arrangement and occupational stress was significant when job autonomy was at high levels, but the relationship was not significant when job autonomy was low. In addition, the results presented in Table 4 show that job autonomy did not moderate the negative relationship between flexible work arrangements and need for recovery as well as for psychological detachment from work. The interaction effects were not significant. Therefore, hypothesis 5 was partly supported.

## Discussion

The present study set out to examine differences in working conditions after a change to open work spaces including ABW on employees’ job demands, job resources, occupational stress, psychological detachment from work and need for recovery. The results revealed relations between flexible working arrangements, occupational stress and need for recovery. The findings indicated potentially beneficial effects of autonomy on employees’ perceived stress levels.

### Differences in job demands and job resources after the transition to open work spaces

We found an increase in perceived workload after the transition to open work spaces. This result was surprising and may be interpreted with the “autonomy paradox” [33]. The paradox is that when workers are given more autonomy and freedom, instead of working less, they work more and longer [33]. In fact, recent studies have shown that flexible working increases work intensity, working hours, and overtime [34, 35]. This type of control over one’s work could have also been given to workers alongside other responsibility, and possibly increased workload as well [36].

We also found a significant difference in perceived job autonomy in that it increased after the transition to open work spaces including ABW. All employees had the flexibility to work at the office or at home. Besides, employees were free in the decision to change their working routines (e.g., work time, location). This might have increased feelings of perceived job autonomy. Medik and Stettina (2014) also reported that most of their study participants associated flexible work arrangements with greater job autonomy and flexibility in working time and location [37]. A meta-analysis by Engelen et al. reported on several studies that show an increase in perceived job control in ABW working environments where employees can decide when and where to do the work [2, 3, 38].

This study also showed significant differences in mental demands after the transition to flexible work arrangements in open work spaces. The transition to ABW seemed to make working somewhat less demanding at least at the longer term. This parallels earlier studies which demonstrated that ABW reduces mental demands and time pressure [1, 7, 39]. An explanation can be found in a study by Kelliher and Anderson (2008) which reports that flexible work designs allow employees to schedule their work in a way that suits their situation best, thereby saving time and energy [40]. Engelen et al. (2019) concluded that ABW can be promoted as providing some benefits for perceptions of the work environment. However, they also require more high-quality research to strengthen the evidence base further and to establish ABW’s effects [2].

In addition, we found that employees experienced higher levels of collaboration between employees one year after the introduction of flexible work arrangements with ABW. Two previously published studies have been found analyzing relations between ABW and collaboration [41, 42]. Robertson et al. found that collaboration was significantly higher after the change to ABW in comparison to the control group [42]. Blok et al. (2012) reported that ABW supported cooperation with colleagues. Negative effects have been discussed in relation to the length employees worked outside the office [34]. The results also demonstrated no significant improvements in satisfaction with communication climate. The systematic review by Engelen et al. (2019) reported several studies that show positive effects on communication [2]. Ten Brummelhuis et al. (2012) reported ABW as positively associated with improved connectivity as well as effective and efficient communication [1]. In addition, a study by De Been et al. (2015) indicated that employees experience more communication, information and knowledge exchange as a result of ABW and open workspaces [33]. However, De Been et al. (2015) stated that satisfaction with communication was lower in flex offices than in combi offices. The authors base the result on the fact that coworkers are easier to find and approach in combi offices than in flex offices. The location of an employee directly affects the possibility of making contact with other colleagues [33].

### Relations between flexible work arrangements and well-being outcomes

We found a significant decrease in perceived occupational stress after the transition to flexible work arrangements in open work spaces. As theoretical assumed the increase of job autonomy in the decision where and when to work had a positive buffering effect on occupational stress perception and need for recovery. This finding is in line with a controlled trail study by Moen et al. (2016) showing that employees who participated in a pilot work flexibility program reported reduced levels of stress and exhaustion than employees within the same company who did not participate. They explained their results by increased job control and autonomy. Importantly, the participating employees were also more efficient and more productive on the job [43].

We did not find support for the assumption that the change would cause less need for recovery over the short and/or long term. As an explanation, it may need more time for flexible work arrangements to have a significant influence on the need for recovery than expected. Previous studies showed small effects on employees’ perceptions of exhaustion or fatigue [1, 44, 45]. Ten Brummelhuis et al. (2012) reported no direct associations between working in an ABW environment and daily exhaustion [1].

The authors explained this result with the assumption that the advantageous effects of being connected to work (intrinsic motivation, absorbation) outweigh disadvantageous [1]. Personality characteristics may also have influenced the study findings. It is possible that the selected employees are open to change, extravert, and like social interactions with others. These employees may value frequent interactions with others, whereby engagement increases while exhaustion decreases [1].

This was one of the first studies to examine possible effects of flexible work arrangements on psychological detachment from work. We found no significant changes in psychological detachment from work after changing to flexible work arrangements in open work spaces. A study by Mellner et al. (2016) reported that working flexible at different places and time is not related to psychological detachment [46]. They concluded that psychological detachment is more likely to be explained by individual personality characteristics than by working environments [46].

The interacting effect of job autonomy on psychological detachment from work could also not be confirmed. An explanation could also be that the individual character of employees plays the most important role. It seems that employees do not need increased job autonomy to perceive a higher level of detachment from work. No comparative studies exist to discuss this result in more detail.

### Strengths and limitations

In order to gain good insight into the effects, it is important to measure the situation before the implementation takes place as well as several times afterwards [47]. Thus, short-term effects caused by the change towards the flexible work arrangement should have disappeared. A strength of this study was its study design with two follow-up measurements. Most previously studies on this topic analyzed only after the transition but not before [48]. This study has several limitations. We did not include a control group, as all employees of the department changed to open work spaces including ABW. So, we cannot fully conclude that the effects were affected by the transition. Other changes in the company might have had an influence on the results. The external validity of the results is limited. We only include one organization in Germany so the findings cannot be generalized to other organizations. In addition, the sample size is relatively small. This may affect the reliability of the study results. Another major limitation of small studies is that they can over-estimate the magnitude of an association. In total, there is nothing wrong with conducting well-designed small studies; they just need to be interpreted carefully. Therefore, it is important not to make strong conclusions.

Furthermore, the discussion of study results has to rely on a limited base of literature. Up to now there is only a small amount of scientific studies on flexible work arrangements such as ABW. As the discussion showed, research results on flexible work arrangements can be adduced for reference, although it must be taken into consideration that there are several preconditions that differ.

### Practical implications and future research

Based on our research findings some practical advices can be highlighted: Organizations should wisely reflect if a change to open work spaces including ABW will achieve the anticipated goals. Overviewing the research findings, ABW might not be the best working surrounding and concept for all employees and job tasks. For job tasks that need high levels of inter-team communication and collaboration, ABW and open workspaces might be valuable. In contrast, job tasks that need high levels of intra-team communication and collaboration should be performed in traditional combi-offices [13]. Building an open work culture with increased autonomy, decision-making, information sharing and collaboration in teams should be a central goal when changing to flexible work arrangements such as ABW [41]. For a successful change, communication is a main factor. Open communication through numerous mediums and employee participation in the process are central success factors [49]. Goal orientated leadership is essential since managing employees is totally different when it is no longer obvious were, when and what employees are working on. Trust in employees is essential for supervisors [50]. They have to focus more on employees’ output instead of presence at the office.

More studies are needed that analyze employees’ working conditions in relation to performance and health-related behavior both before and after transferring to flexible work arrangements in open work spaces. Field studies that proved the relationships of this study would be highly valuable. In addition, longitudinal research should include a control group. Studies performed in laboratories might be an alternative because they allow for higher control but limit generalizability due to artificial settings and study participants.

## Conclusions

The findings of this study provide new information on effects of a transition to open work spaces including ABW on employees’ job demands, job resources and well-being outcomes. The study showed some beneficial effects in reducing mental job demands and in increasing several job resources such as job autonomy. Overall, these findings seem to indicate that flexible work arrangements are related to some positive effects on employees’ perceived occupational stress, psychological detachment from work and need for recovery. However, we are aware on the methodological limitations that might have been influencing the results.

This study built a basis for future longitudinal research on the effects of flexible work arrangements in open work spaces. Future research studies are needed to investigate different relations between flexible work arrangements such as ABW and job performance as well as health-related outcomes.

## Data Availability

The data analyzed during the current study are not publicly available due to German national data protection regulations.

## Abbreviations

ABW: Activity Based Working
F: F-test value
M: Mean
RM ANOVA: Repeated measures analysis of variance
RM MANOVA: Repeated measures multivariate analysis of variance
SD: Standard Deviation
